# The use of muscle strength assessed with handheld dynamometers as a non-invasive biological marker in myotonic dystrophy type 1 patients: a multicenter study

**DOI:** 10.1186/1471-2474-11-72

**Published:** 2010-04-18

**Authors:** Luc J Hébert, Jean-François Remec, Joanne Saulnier, Christophe Vial, Jack Puymirat

**Affiliations:** 1 Canadian Forces Health Services Headquarter, Directorate Medical Policy, National Defense of Canada, 1745 Alta Vista Dr, Ontario (K1A 0K6), Ottawa, Canada; 2 Department of Radiology, Faculty of Medicine, Laval University, Pavillon Vandry (room 3370), Quebec (G1K 7P4), Quebec, Canada; 3 Service de neuro-rééducation, 59 boulevard Pinel, 69677 Bron cedex, Lyon, France; 4 Institut de réadaptation en déficience physique de Québec, 525, boulevard Wilfrid-Hamel, Quebec, (G1M 2S8), Quebec, Canada; 5 Department of Electro-neurophysiology and muscular pathology, Hôpital Pierre Werteimer Groupement Hospitalier Est, 59 boulevard Pinel, 69677 Bron cedex, Lyon, France; 6 Human Genetic Research Unit, Centre Hospitalier de l'Université Laval, 2705 boulevard Laurier, Quebec (G1V 4G2), Quebec, Canada

## Abstract

**Background:**

Myotonic dystrophy type 1 (DM1) is a multisystem disorder that demonstrates variable symptoms and rates of progression. Muscle weakness is considered one of the main problems with a clinical picture that is characterized by distal weakness of the limbs progressing to proximal weakness. The main objective of this study was to characterize the maximal strength of ankle eversion and dorsiflexion in DM1 patients. Manual and handheld dynamometer (HHD) muscle testing were also compared.

**Methods:**

The maximal strength of 22 patients from Quebec (mean age = 41,1 ± 13,8) and 24 from Lyon (mean age = 41,6 ± 10,2) were compared to 16 matched controls.

**Results:**

With the use of HHD, an excellent reproducibility of the torque measurements was obtained for both centers in eversion (R^2 ^= 0,94/Quebec; 0,89/Lyon) and dorsiflexion (R^2 ^= 0,96/Quebec; 0,90/Lyon). The differences between 3 groups of DM1 (mild, moderate, severe) and between them and controls were all statistically significant (p < 0,001). No statistical differences between sites were observed (p > 0.05). The degree of muscle strength decline in dorsiflexion (eversion) were 60% (47%), 77% (71%), and 87% (83%) for DM1 with mild, moderate, and severe impairments, respectively. The smallest mean difference between all DM1 patients taking together was 2.3 Nm, a difference about twice than the standard error of measurement. There was a strong relationship between eversion and dorsiflexion strength profiles (R^2 ^= 0,87;Quebec/0,80;Lyon). Using a 10-point scale, manual muscle testing could not discriminate between the 3 groups of DM1 patients.

**Conclusions:**

The HHD protocol showed discriminative properties suitable for multicentre therapeutic trial. The present results confirmed the capacity of quantitative muscle testing to discriminate between healthy and DM1 patients with different levels of impairments. This study is a preliminary step for the implementation of a valid, reliable and responsive clinical outcome for the measurement of muscle impairments with this population.

## Background

Myotonic dystrophy type 1 (DM1) is an autosomal dominant disorder that is the most common inherited myopathy in adults[1]. In Quebec (Canada), a prevalence of 189 per 100,000 population has been reported in the Saguenay-Lac-Saint-Jean region, which is more than 13 times the highest population world-wide prevalence reported [1,2]. DM1 is a multisystem disorder that demonstrates variable symptoms and rates of progression. Muscle weakness is considered one of the main problems with a clinical picture that is characterized by distal weakness of the limbs progressing to proximal weakness. The progression of a general weakness of all lower limb muscles, often worse in the distal muscles, will lead to reduce balance and bilateral foot drop. Indeed, people with DM1 often present with weakness of the ankle dorsiflexor muscles that may lead to falls due to tripping [3,4].

Previous studies with myotrophic dystrophy have characterized muscle strength deficits, established baseline data and compared the rate of change in strength of proximal and distal muscles. Different tools were used to assess the muscular impairments including manual muscle testing, timed functional tests, quantitative motor evaluation, isokinetic systems, Jamar dynamometer, and hand held dynamometers [2-7]. From those studies, few consensuses have emerged. Distal weakness first appears and is usually identified after 9 to 10 years' duration of the illness. There is a faster decline for distal than for proximal muscles, and functional activities such as gait are rapidly affected and lead to a significant level of disability. The rate of change in muscle strength during the natural course of disease or during rehabilitation is significant but slow and may be difficult to detect using manual muscle testing or ordinal scales. Manual muscle testing, even when using reliable scales, lacks sensitivity to detect subtle changes in muscular impairment and is not appropriate to measure the efficacy in short-term therapeutic trials, and neither suitable for longitudinal studies for which a discriminatory measure is required [8].

Hand held dynamometer (HHD) protocols represent a good option for quantitative muscle testing in a clinical setting. HHDs are portable, economic, and user-friendly. The use of HHD provides a quick, simple, valid, reliable and sensitive outcome measurement of the human muscle strength and a high level of agreement can be obtained with this type of quantified muscle testing [9-15]. Mathieu et al. (2003) used a Nicholas push HHD to measure the maximal isometric force of elbow extension, hip flexion, and ankle dorsiflexion [2]. In a previous work, we developed a standardised protocol to assess the maximum isometric torque (MIT) with the use of a HHD for several muscle groups of both the lower and upper limbs. The feasibility, intra- and inter-rater reliability, and concurrent validity of this protocol with the Cybex dynamometer were found to be very good to excellent (Hébert LJ et al., World Congress of Physical Therapy, Vancouver, BC, Canada, June 2007). Also, the concurrent validity of the HHD protocol with the Cybex was good to very good with ICCs values of 0.79 (evertors) and 0.84 (dorsiflexors).

In view of future therapeutic trials for DM-1 patients, ankle dorsiflexor and evertor muscles could be used to monitoring the efficacy of treatment. These muscles are affected early in the disease progression and are easy to inject and analyze. Also, as the evertors are the best protection for a near-maximally inverted ankle at footstrike, they act in synergy with the ankle dorsiflexors to stabilise the foot-ankle complex during weight bearing activities and they may compensate for a lack of dorsiflexion [16]. In view of future therapeutic trials for DM1 patients, ankle dorsiflexor and evertor muscles could be used to monitoring the efficacy of gene therapy following systemic or multisite intramuscular injections of either viral vectors or antisense oligonucleotides. Therefore, in the current study, both the ankle evertors and dorsiflexors were considered.

The objectives of this study were to characterize the maximal isometric strength of ankle evertors and dorsiflexors with HHD quantitative muscle testing in DM1 patients to establish the intra- and inter reliability, which is of major importance for future multicentric therapeutic trials. Manual and quantitative muscle testing were also compared to provide data to validate the most appropriate method for monitoring muscular changes in patients with DM1 presenting with a large spectrum of impairments.

## Methods

### Participants

Inclusion criteria were limited to DM1 patients older than 18 years old, weakness of the tibialis anterior (TA) as assessed by manual muscle testing, DM1 diagnosis confirmed by molecular testing, and to the patient' ability to take part in manual and quantified muscle strength assessment. Patients with congenital form of the disease, cognitive impairments, Body Mass Index (BMI) > 30, pregnant women, and severe pulmonary deficiencies were excluded from the study. Patients with congenital or juvenile DM, severe pulmonary deficiencies (FVC < 50%) and severe sleepiness were excluded: the latter two groups were excluded because they would not have been able to undergo the full research protocol. Patients were recruited from the medical chart based on the manual testing scores of the TA to ensure a wide distribution of muscle impairment from mild (≥ 4 et < 5), to moderate (≥ 3 et < 4), and severe (< 3). At the inclusion in the study, all DM1 patients and controls recruited were tested using the conventional 0-5 manual muscle testing scale. Thereafter, using the Modified Research Council Scale (MRCS) with a 0 to 10 point scoring system, all patients were assessed by the physiotherapist and classified according to the score obtained in: Group 1 (G1, mild) for a score from 7 to10, Group 2 (G2, moderate) for a score from 4 to 6, and Group 3 (G3, severe) for a score from 0 to 3 [2].

A sample of convenience of 22 patients (M/F 11/11) in Quebec and 24 patients in Lyon (France) (M/F 11/13) with different levels of muscle impairments participated in the study. A random probabilistic sample of 16 control subjects (G0) was established using the members of the patient family as paired subjects for age and gender. For all controls, the molecular testing was negative, they were not affected by any muscular pathology.

The project was approved by the ethic committees of the IRDPQ for Quebec and of the Cardio-neurology Hospital for Lyon. Written informed consent was obtained from all patients and controls prior to the examination.

### Procedures

#### Manual muscle testing

A physiotherapist using manual muscle testing assessed the maximum isometric muscle strength of the ankle dorsiflexors and evertors. Both muscle groups were assessed with the subject sitting on the edge of a table, both legs hanging, the ankle at 90° of flexion. The subject was first asked to do a full dorsiflexion or an eversion of the ankle as far as possible. At the end of the movement, the examiner was doing a break test trying to break the resistance toward the plantar flexion (to assess the dorsiflexors) or inversion (to assess the evertors). For both muscle groups, a modified version of the modified MRCS (MM-MRSC) with a 0 to 10 point scoring system was used to quote the muscle group [5]. The lowest score obtained was used to classify the subject. The same physiotherapist was completing both manual and quantitative muscle testing within the same session. The definitions of the individual muscle testing grades and corresponding scores of the MM-MRSC used are presented in Additional file 1.

#### Quantitative muscle testing

**Figure 1:**
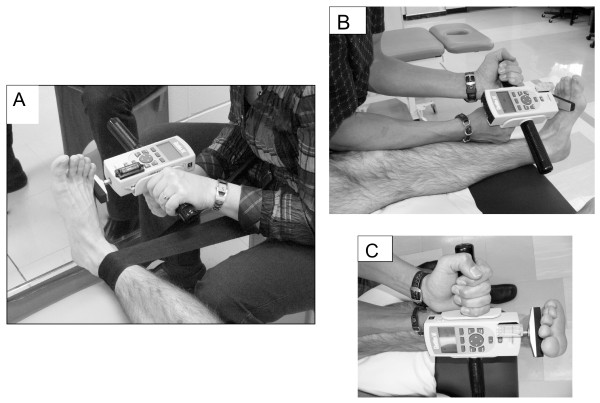
**The maximum isometric muscle strength of the ankle evertors (A) and dorsiflexors (B and C) was assessed with a Chatillon push-pull hand-held dynamometer**. The positioning of the subject was standardised ensuring that «gravity eliminated» positions were used for each muscle group.

The new generation of push-pull HHDs are more accurate and sensitive and they offer a better ergonomic design for the examiner to resist different muscle groups in several alternative positions. The maximum isometric muscle strength of the ankle dorsiflexors and evertors was assessed with a Chatillon push-pull hand-held dynamometer (FCE-500, Ametek TCI Division, Chatillon Force Measurement Systems, Florida, USA). All physiotherapists received an extensive training on the use of the standardized HHD protocol. The Figure 1 shows the positioning of the subject that was standardised to ensure that «gravity eliminated» positions were used for each muscle group [14]. Both ankle dorsiflexors and evertors were tested with the subject supine with a small roll under the knee to allow 5-10° degrees of knee flexion, ankle maintained at 90° of flexion. Isometric make tests were used because they are more reliable[17,18], more comfortable for the patient, and have shown a lower risk for injury compared to break tests[10]. Standard verbal encouragement was given during each maximal contraction. Two trials were completed for each muscle group, and the mean of the two trials was used for the final analysis. When more than 10% difference was noted between the two trials, a third one was completed. The units of force in Newton were multiplied by the corresponding lever arm, measured in metre, to calculate the maximal isometric torque in Newton-meters (Nm).

Before each trial, the subject was asked to perform two sub-maximal contractions of about 50% to warm-up, ensure the task was well understood and verify that the stabilisations were adequate. Subjects were instructed to avoid explosive contraction and to increase their effort gradually to their maximum immediately after hearing the signal «3,2,1, GO». The tone and words of encouragement used by the tester were also matching a crescendo pattern to make sure the maximum contraction was produced at every trial. Each contraction was held 10 seconds (the dynamometer was recording the peak force) maximal force was followed by a 60 second-rest period. Due to the lower level of endurance and fatigue of some patients, additional and sufficient resting time was given before proceeding to a second trial. Strength measurements were tested bilaterally and values were averaged for the two sides. At baseline, strength was measured at day one (D1) and 21 days later (D21) by one experienced physiotherapist (one in Quebec and one in Lyon). When testing at D21, the tester was blind about the subject's strength values obtained at D1.

#### Preliminary testing for reliability

Even though the HHD protocol used has shown excellent psychometric properties, the specific inter-rater reliability of the evaluators of the current study was verified. Three examiners, the two experienced physiotherapists appointed to the study at each centre and one backup for the Quebec site, assessed 9 healthy subjects with the above HHD protocol. The inter-rater reliability was then calculated using Pearson correlation coefficients. The above protocol was used as described except that 5 trials per muscle group were completed.

#### Data analysis

Descriptive statistics (mean and standard deviations) of maximal torques were calculated for ankle evertors and dosrsiflexors of patients of all groups and controls. Paired t-tests were used to verify significant statistical differences between groups and centres. Simple linear regressions were used to determine the relationships between the maximal strength between evertors and ankle dorsiflexors for both centres (Lyon and Quebec). Maximal torques obtained with the HHD quantitative muscle strength assessment were plotted against the scores obtained with the manual muscle strength assessment from the MMRC to verify the discriminative properties of manual muscle testing. The original alpha level for the statistical analyses was set at 0.05.

## Results

Prior to the study, preliminary testing using the above-described HHD protocol was done. Three physiotherapists assessed a total of nine healthy subjects over two sessions. Five trials per muscle group per side were completed. Results showed a good to excellent inter-rater reliability between all testers with Pearson correlation coefficients (r_p_) ranging from de 0.70 to 0.93 and 0.72 to 0.94 for ankle dorsiflexion and eversion, respectively. Also, the Standard Error of Measurement (SEM) was 1.0 Nm for the dorsiflexion, and 1.3 Nm for eversion.

The 22 patients (11 males and 11 females; 11 in G1, 6 in G2, and 5 in G3) from Quebec and the 24 from Lyon (11 males and 13 females; 11 in G1, 6 in G2, and 7 in G3) who participated to the study had a mean age of 41,1 ± 13,8 (patients from Quebec) and 41,6 ± 10,2 (patients from Lyon). Most of patients had myotonia but did not receive any drugs. The matched control group was composed of 16 healthy subjects including 7 persons (5 males and 2 females) from Quebec with a mean age of 43,1 ± 9.0, and 9 persons (7 males and 2 females) from Lyon with a mean age of 45,7 ± 9,0. In the present study, the mean number of years of disease duration were 19.2 (G1), 30.2 (G2) and 26.6 (G3).

**Figure 2:**
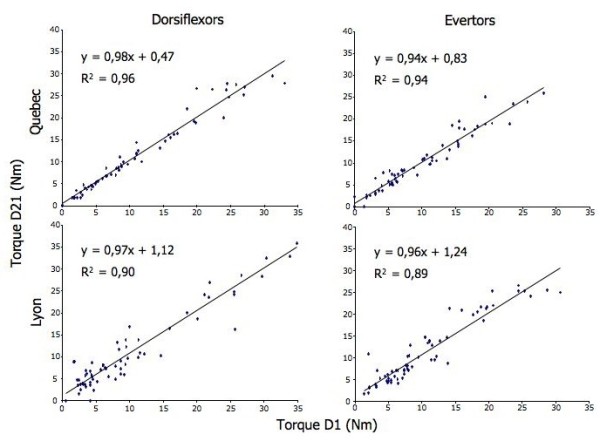
**Reproducibility of muscle strength at baseline between Day 1 and Day 21 for the ankle evertors and dorsiflexors for Quebec (n = all trials of 21 patients and 7 controls) and Lyon (n = all trials of 24 patients and 9 controls)**. The equation and coefficients of determination R^2 ^are indicated for each muscle group and both centres.

As observed on the Figure 2, an excellent reproducibility of the torque measurements at baseline were obtained between Day 1 and Day 21 for both centers in eversion (R^2 ^= 0,94 for Quebec and 0,89 for Lyon) as well as in ankle dorsiflexion (R^2 ^= 0,96 for Quebec and 0,90 for Lyon).

**Figure 3:**
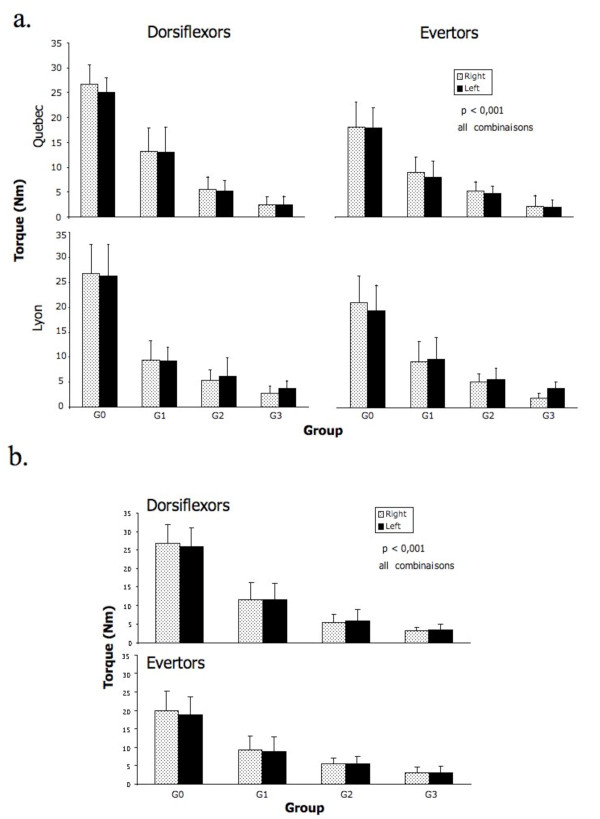
**a and b. The top figure shows the mean peak torque and SD of ankle evertors and dorsiflexors of controls (G0) and DM1 patients as classified into Group 1 (G1, mild), Group 2 (G2, moderate), and Group 3 (G3, severe) according to the severity of their muscle impairment**. The bottom figure illustrates the same data pooled for the two study sites.

The torques values of ankle evertors and dorsiflexors of all groups for both centers are reported in detail in the Table 1. The same data is graphically reported and shown in Figure 3a. The differences between the three DM1 groups as well as between the DM1 patients and the controls were all statistically significant (p < 0,01). However, when comparing the data between Lyon and Quebec, no statistical differences between sites for each group were observed (p > 0.05). Therefore, the pooled data at baseline for both sites is reported in Figure 3b. As observed on this Figure, the progression of muscular impairment in ankle dorsiflexion (eversion) demonstrates a degree of muscle strength decline of 60% (47%), 77% (71%), and 87% (83%) between the control subjects and the patients with mild, moderate, and severe impairments, respectively.

**Table 1:** Torque values ± SD* in Newton-meters of ankle evertors and dorsiflexors of all groups (n = number of subjects tested) for both centers.

	DM1 - Group 1 (Quebec n = 11) (Lyon n = 11)		DM1 - Group 2 (Quebec n = 6) (Lyon n = 6)		DM1 - Group 3 (Quebec n = 5) (Lyon n = 7)		Control (Quebec n = 7) (Lyon n = 9)	
	
Dorsiflexion	right	left	right	left	right	left	right	left
Quebec	12.8 ± 3.7	12.7 ± 4.9	6.9 ± 2.4	6.3 ± 1.9	3.2 ± 1.1	3.2 ± 1.5	25.8 ± 6.0	24.6 ± 4.6

Lyon	9.4 ± 3.8	9.2 ± 2.8	5.3 ± 2.2	6.2 ± 3.7	2.7 ± 1.5	3.8 ± 1.4	26.8 ± 5.8	26.3 ± 6.2

Eversion								

Quebec	12.3 ± 3.8	12.5 ± 5.3	6.0 ± 2.2	6.0 ± 1.7	5.0 ± 2.5	3.8 ± 2.9	19.6 ± 5.1	18.5 ± 3.9

Lyon	8.9 ± 3.2	8.0 ± 3.2	5.2 ± 1.8	4.7 ± 1.5	2.1 ± 2.2	1.8 ± 1.5	18.1 ± 5.0	17.9 ± 4.2

The differences between the three DM1 groups as well as between the DM1 patients and the controls were all statistically significant (p < 0,001). However, when comparing the data between Lyon and Quebec, no statistical differences between sites for each group were observed (p > 0.05).

* Standard Deviation

**Figure 4:**
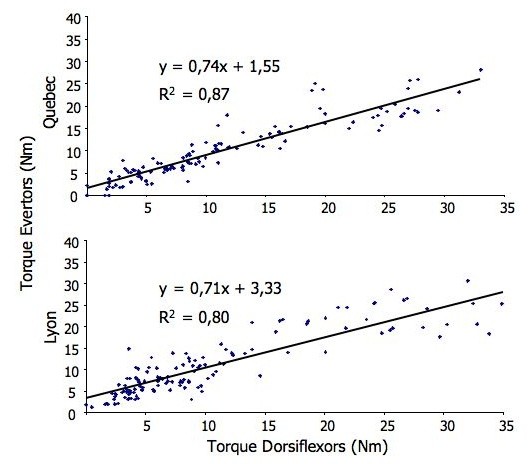
**Relationships between the maximal strength (Day 1 and D 21 values combined; n = 180 trials) of the ankle evertors and dorsiflexors for Quebec and Lyon as estimated with simple linear regression**. The equation and coefficients of determination R^2 ^are indicated for each centre.

As shown on Figure 4, for both centres, there is a strong relationship between eversion and ankle dorsiflexion peak torques obtained with the HHD with coefficients of determination (R^2^) of 0,87 and 0,80 for Quebec and Lyon, respectively.

**Figure 5:**
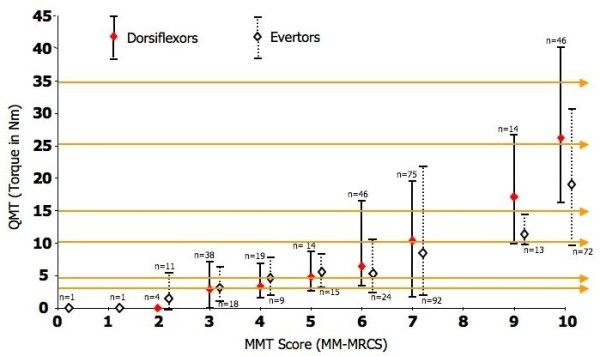
**Correspondence between the peak torques for the evertor (dotted lines) and dorsiflexor (continuous lines) muscles obtained with quantitative muscle testing (QMT) and manual muscle testing (MMT) scores obtained using the Modified-modified medical research council scale (MM-MRCS)**. For each MMT score from 0 to 10, the number of trials used (n) for comparisons is indicated. The horizontal arrows are few examples that show, for successive peak torque of 2.5, 5, 10, 15, 25, and 35 Nm, the numerous corresponding MMT scores that could be obtained according to the QMT. The empty and full diamonds in line with each MMT score indicate the mean QMT value, and the related two-tail T bar line the highest and lowest values for that specific MMT score.

The Figure 5 illustrates the correspondence of peak torques for all DM1 patients obtained with MMT and QMT. For each patient, the results of his MMT and QMT scores were plotted one against each other. As observed, different patients that had different levels of muscle strength according to the 10 point-scale MMT on the X axis produced in fact the same torque as indicated by the QMT values on the Y axis. Several examples are provided for strength values ranging from 2.5 to 35 Nm, a magnitude of strength that corresponds to the range of strength we would observe with DM1 patients. The weaker the patients (the lower the QMT peak torques), the more important the possibility of misclassifying a patient (the more important the number of possible MMT scores). For example, the horizontal arrows at the 2.5 and 5 Nm values on the Y axis are crossing 5 different point scale of muscle strength, from 2 to 7, as assessed with MMT.

## Discussion

The use of a valid and reliable protocol to detect low sub-maximal muscle impairments in DM1 patients is essential to characterize the natural history of muscular involvement and measure the efficacy of short-term therapeutic trials in multicenter studies. The method used to assess muscle strength must be simple, easy to perform for the clinician and it must be sensitive enough to allow the early detection of distal muscle weaknesses. Moreover, the method used must be able to discriminate between different levels of severity of muscle impairments for choosing the best clinical decisions with regard to treatment plan.

This study has allowed us to characterize the maximal isometric strength of ankle evertors and dorsiflexors with quantitative muscle testing between three levels of severity of DM1 (mild, moderate and severe) and control subjects in a multicenter study setup. When averaging the torque values for genders, sides, centers, and days at baseline, the mean peak torque of the control subjects was 26.5 Nm. This is similar to the values reported by Hogrel et al. (2007) that was 30.5 Nm when combining men and women[19]. The slight difference observed between studies could be explained by methodological differences. The values from Hogrel et al. were reported for a 20-80 year old range compared to a 40-50 decade in our study, and ankle dorsiflexion measurements were performed with the knee flexed and against gravity. Bäckman et al (1995) and Hasegawa et al. (2008) reported HHD strength values for the ankle dorsiflexors for healthy subjects for the 40-50 decade of age but unfortunately, the units used in these studies were the Newton and Newton/Kg, which does take into account the lever arms and does not allow a proper comparison [20,21].

Our study suggests that the initial decline of muscle strength in DM1 patients in the first 19 years of the disease (comparing the change of strength between G0 and G1) is about 2.5% (evertors) to 3.2% (dorsiflexors) per year followed by a rate of progression of about 1.5% (dorsiflexors) to 2.2% (evertors) for the subsequent 11 years. Using a Nicholas push HHD, Mathieu et al. (2003) previously reported a strength decline per year of disease duration of 1.2-1.6% for hip flexors, a proximal group, and 2.0-3.0% for the hand grip flexors, a distal group[2]. These results concur with our study and suggest that the lost of strength in distal muscles is progressive and might be as important for both the upper and lower limbs.

Because our study design was not longitudinal, it is not possible to know if the progression of the muscle strength decline is linear or not. Also, as the disease duration is assessed essentially through the patient's interview, it remains an estimate which may not be perfectly accurate.

In the current study, both the ankle evertors and dorsiflexors were considered. The justification to include the evertors in the current study was based on the premise that, near-maximally inverted ankle at footstrike, the evertors would act in synergy with the ankle dorsiflexors to stabilize the foot-ankle complex during weight bearing activities. Because of the consistent muscle weakness of ankle dorsiflexors reported in the literature in DM1 patients, we hypothesised that the evertors could compensate for a lack of ankle dorsiflexion strength. However, the current findings suggest that there is no added value to measure the strength of both the evertor and dorsiflexor muscles to follow the progression of the disease in DM1 patients.

The HHD protocol used showed an excellent reproducibility of the measures at baseline even when the protocol was administered by different centers and testers. This later finding confirms the usefulness of using a HHD protocol for longitudinal follow-up in a future multicentre therapeutic trial aimed at assessing the efficacy of a gene therapy. The present results also confirmed the capacity of QMT to discriminate between healthy and myotonic dystrophy type 1 patients with different levels of impairments. Even when manual muscle testing is used by clinicians who have several years of experience and are utilizing a more sensitive tool such as the MM-MRSC 10 point scale, as shown in the present study, it cannot properly classify DM1 patients. As seen on Figure 5, subjects with the same peak torque would have been attributed a score of 2, 3, 4, 5, 6, and even 7. This misclassification being worse for weaker patients, it may have a significant negative impact on the clinical decision making. A DM1 patient with a score of 7 with MMT would have been considered as having a mild muscular impairment while in fact, using the QMT, he may have had a very low torque and would therefore have been considered as having severe muscle impairment. In this case, this may have led to classify the patient in a different DM1 group and may also have led to different clinical recommendations. This is in agreement with Whittaker et al. (2006) who concluded that MRC scale is unsuitable for detecting the small changes in strength seen in a slowly progressive disease such as myotonic dystrophy[22]. In fact, the mean torque difference between the control group and the DM1 patients was ranging from 8.1 to 22.7 Nm. Also, the smallest mean group difference observed between DM1 patients was 2.3 Nm (between G2 and G3 in eversion), a difference about twice than the SEM. Therefore, using a HHD protocol, one can be confident that even small between group muscle strength differences observed might be associated to a true physiological change and not only the result of measurement errors.

In addition to verify the feasibility of measuring the isometric muscle strength assessment with DM1 patients using a hand-held dynamometer protocol, this study is a preliminary step for the development of a valid, reliable and responsive outcome for the measurement of muscle impairment with this population. This measure could be used to monitor the major stages of DM1 progression, characterize the natural history of the disease, verify the efficacy of therapies, and document the relationship between impairments and disabilities. However, future research should be done to better characterize the inter-testers reproducibility using our method and to look at systematic differences between sessions for other muscle groups. The present results are very promising and provide us with the basis we need to move forward and proceed with a longitudinal study to determine the rate of muscle strength decline through all stages of the disease and to verify the potential of quantitative measures of muscle impairments as a valid indicator of other biological changes.

## Conclusions

Manual muscle testing, even when used by experimented clinicians, cannot be used to properly classify DM1 patients. Our results confirm the usefulness of using a standardized HHD protocol to measure muscle strength in DM1 patients. The capacity of an HHD protocol to discriminate between healthy and DM1 patients with different levels of impairments suggests that this method may be used as a biological marker for longitudinal follow-ups.

## Competing interests

The authors declare that they have no competing interests.

## Authors' contributions

LJH participated in the design of the study, carried out the acquisition, the analysis and the interpretation of data, and he drafted the manuscript. JFR participated in the design of the study, in the analysis and interpretation of data, and he has been involved in drafting the manuscript. JS participated in the design of the study, in the analysis and in the interpretation of data, and he has been involved in drafting the manuscript. CV participated in the design of the study, in the analysis of data, and has been involved in drafting the manuscript. JP participated in the design of the study, the acquisition, the analysis, and in the interpretation of the data, and he has also been involved in drafting the manuscript. All authors read and approved the final manuscript.

## Authors' information

Dr Jack Puymirat (JP) is the Director of the French Canadian program on Myotonic dystrophy supported by AFM.

## Pre-publication history

The pre-publication history for this paper can be accessed here:

http://www.biomedcentral.com/1471-2474/11/72/prepub

## Supplementary Material

Additional file 1 **Appendix**. Modified-modified medical research council scale (MM-MRCS). Click here for file
