# Supplementary material for: The use of muscle strength assessed with handheld dynamometers as a non-invasive biological marker in myotonic dystrophy type 1 patients: a multicenter study
Source: BMC Musculoskelet Disord. 2010 Apr 18;11:72. doi: 10.1186/1471-2474-11-72 (PMC2868792; doi:10.1186/1471-2474-11-72)
Supplement: Additional file 1 — Appendix. Modified-modified medical research council scale (MM-MRCS). [file 1471-2474-11-72-S1.DOC]

Appendix 1. Modified-modified medical research council scale (MM-MRCS)

Manual muscle strength was assessed and scores were assigned using a 10-point MM-MRCS. Definitions of the individual muscle testing grades and corresponding scores were:

| **Grade** | **Modified-Modified Medical Research Council Scale** | **Score** |
| --- | --- | --- |
| 5 | Normal strength | 10 |
| 5- | Barely detectable weakness | 9 |
| 4+ | Same as grade 4, but muscle holds the joint against moderate to maximal resistance | 8 |
| 4 | Muscle holds the joint against a combination of gravity and moderate resistance | 7 |
| 4- | Same as grade 4, but muscle holds the joint only against minimal resistance | 6 |
| 3+ | Muscle moves the joint fully against gravity and is capable of transient resistance, but collapses abruptly | 5 |
| 3 | Muscle cannot hold the joint against resistance, but moves the joint fully against gravity | 4 |
| 3- | Muscle moves the joint against gravity, but only through the ¾ of the full mechanical range of motion | 3 |
| 2+ | Muscle moves the joint against gravity, but only through the 1/3 of the full mechanical range of motion | 3 |
| 2 | Muscle moves the joint fully when gravity is eliminated | 2 |
| 2- | Muscle moves the joint only through the 1/3 of the full mechanical range of motion when gravity is eliminated | 2 |
| 1 | A flicker of movement is seen or felt in the muscle | 1 |
| 0 | No movement | 0 |
